# Sloughing of Twenty-six Inches of Intestine

**Published:** 1830

**Authors:** 


23. *Sloughing of twenty six inches of Intestine.* In the Journal just referred to, Dr. Gaylord of Sodus, N. Y. has reported a case of intus-susception, in which a portion of intestine, measuring about 26 inches, sloughed off and was discharged *per anum.* The patient became extremely emaciated afterwards, and was affected with flatulence and intestinal spasms for a long time. A gentle mercurial salivation at length subdued these affections; and a state of tolerable health ensued, interrupted, however, by an occasional attack of colic.

